# Newborn Screening Services in Bahrain between 1985 and 2010

**DOI:** 10.1155/2012/903219

**Published:** 2012-04-08

**Authors:** Shaikha Al Arrayed, Amani Al Hajeri

**Affiliations:** Genetic Department, Salmaniya Medical Complex, P.O. Box 12, Bahrain

## Abstract

*Introduction*. The incidence of genetic blood disease in Bahrain has declined gradually since 1984 when the Ministry of Health (MOH) instituted a prevention campaign. The national NBS program for hemoglobinopathies was started in May 2007, financed by the national budget. *Setting*. Genetics department, Salmaniya Medical Complex, MOH, Bahrain. *Methodology*. The genetics, nursing, pathology, and pediatric departments were involved in the study. This service was offered to all infants. Cord blood samples were collected at birth and were then sent to the laboratory. *Results*. During 3.5 years after the program was implemented, we screened 38,940 newborns (NBs), of which 17,375 were screened in 2008, 10,248 in 2009, and 11,317 in 2010. The number of affected NBs was 128 in both 2007 and 2008, 58 in 2009, and 47 in 2010, as the average number of affected NBs in 2010 was 4 per month. The incidence of affected NBs was found to be 0.7% in 2008, 0.6% in 2009, and 0.4% in 2010. *Conclusion*. NBS is an essential step for the early diagnosis and treatment of affected NBs, future recurrence of the disease in the same family. In Bahrain, the number of affected NBs has declined by 75% during the last 20.

## 1. Introduction

Bahrain comprises an archipelago of 36 islands, with an area of 717.5 km^2^, located in the Arabian Gulf. Saudi Arabia is situated to the west and Qatar to the east. The estimated population of Bahrain in 2010 was 1,039,297. The 2001 census data showed that the population of Bahrain was 646,551 in 2007, of which Bahraini nationals constituted 50.7% and non-Bahrainis constituted 49.3%. Most Bahraini Arabs are originally from the Arabian Peninsula. The crude birth rate was estimated to be 15.4/1000 individuals, whilst the infant mortality rate was 83/1000 live births in 2007 [3].

Genetic blood diseases (GBDs) are common in Bahrain and in all Middle Eastern countries [4, 5]. Bahrain is the first country in the Middle East region to tackle this issue since 1984 when the Ministry of Health (MOH) of Bahrain recognized the importance of controlling these diseases. In 1984, the first genetics clinic was established, which began educational campaigns. Information booklets were prepared and distributed widely in an attempt to increase awareness about these diseases among the public.

The campaigns, which have been conducted since 1980, focused on public awareness, education, and screening pertaining to antenatal issues and premarital services (1993), students and premarital issues (1998), newborns (2007), and prenatal testing [6].

In 1991, the Bahrain Hereditary Anemia Society was formed with the aim of promoting awareness among the people of Bahrain. Molecular laboratories have been established in the main hospitals to study genotype-phenotype correlations, and the mutations that cause GBDs have been characterized.

We obtained support from religious scholars, and the parliament. In 2004, the king of Bahrain issued a law of premarital counseling. In 2007, the ministerial cabinet approved the budget for the newborn screening (NBS) program.

Several teams were involved in the campaign, including clinical geneticists, primary health care workers, pediatricians, hematologists, molecular geneticists, school teachers, students, religious scholars, policymakers, nurses, and technicians.

The campaign continued for approximately 20 years. Various issues related to ethical, legal, and social implications (ELSI), such as informed consent, privacy, equity, confidentiality, and prevention of stigmatization and discrimination, were addressed during the screening programs.

The NBS program is a well-recognized health program aimed at early identification of infants who are affected by certain genetic, metabolic, or infectious conditions. Early identification of these conditions is particularly crucial as timely intervention can significantly reduce morbidity, mortality, and associated disabilities in affected infants and save thousands of babies from mental retardation, death, and other complications.

This paper describes the NBS program for sickle cell disease (SCD) and sickle cell thalassemia in Bahrain and analyzes the results of the last 3.5 years of screening service.

## 2. Material and Methods

The screening is offered to all infants who are delivered in the MOH maternity units. An explanatory leaflet detailing the purpose, process, and outcomes of the screening service was provided to the parent(s) by midwives before screening. The decision to opt out of testing was documented.

A questionnaire for each newborn was completed by a nurse. The form included data regarding demographic characteristics, parental age group, and consanguinity and was coded for computerization.

## 3. Sample

The cord blood of the infants was collected at birth and posted to the central laboratory for testing.

Sample analysis: high-performance liquid chromatography according to Eastman et al. 1996 [6] was used to perform electrophoresis on the cord blood samples to detect the hemoglobin fractions present, and a fluorescent screening technique, according to Jiang et al. 2003 [7], was used to detect glucose-6-phosphate dehydrogenase (G6PD) deficiency. In cases where an abnormal band was suspected, a second-line test isoelectric focusing was performed to confirm the presumptive diagnosis. Hematologists interpreted the results, and, in few cases, diagnosis could be achieved only by using DNA analysis. All the results obtained were sent by the laboratory technicians to the genetics department where further action to be taken depended on the categories of the results as follows.

 For NBs with SCDs or any other hematological conditions, which required follow-up, the parents were informed by personal contact and they were asked to visit the clinic, where a confirmatory test was performed. The newborn was referred to a pediatrician for management and follow-up if the suspected condition was confirmed.

The infant's family was referred to the maternal and child health facility if no abnormality was detected or if the infant was heterozygous for a hemoglobin variant.

## 4. Results

The number of affected newborns per month in 2010 is shown in Table 1, while the number and percentage of affected newborns per year during 2007–2010 are shown in Table 2.

**Figure 1:**
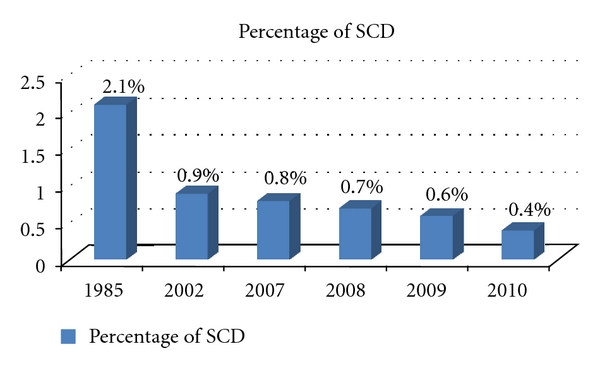
Decline in the newborns incidence of sickle cell disease between 1985 and 2010.

The decline in the incidence of SCD between 1985 and 2010 is shown in Figure 1.

## 5. Discussion

The MOH in Bahrain recognized that NBS is an essential step toward the early diagnosis and treatment of genetic diseases among newborns, for avoiding further complications and for advising parents so as to prevent future recurrence of these diseases in the same family.

Many pilot studies were performed before starting the program at the national level. The results of these studies mandate this program because of the high rate of hemoglobinopathies in the community [8].

We tried to implement all the factors necessary for the success of a national NBS program such as making NBS a government priority, providing funding for NBS, ensuring general public acceptance, and ensuring the cooperation of all health practitioners and family physicians.

The screening service started in mid-2007, after obtaining approval from the MOH and allocation of the national budget. Many departments cooperated for implementing the program; it involved the genetics department, pathology department, maternal and child department, nursing department, and the health information directorate.

The senior staff trained nurses and technicians for collecting and testing blood samples.

The NBS program can be considered as an audit for the previous campaign that dealt with controlling genetic diseases. Every newborn affected was studied to find whether he/she belonged to a family with a previous case of SCD or to a new family, and advice was given regarding future pregnancy and care for the affected newborn.

We noticed a gradual reduction in the number of affected newborns, as shown in Figure 1, from 2.1% in 1985 to 0.4% in 2010. This may indicate a gradual decline in disease frequency, which was most probably due to the increased awareness among the people of Bahrain that was a result of the educational and awareness campaign started approximately 20 years ago.

A previous molecular study showed that approximately 90% of patients had a mild form of SCD. DNA analysis has proved the presence of the Asian haplotype as the common haplotype. This mutation is known to be associated with a high level of fetal hemoglobin (HbF), a situation similar to that in the eastern province of Saudi Arabia [7, 9–11]. 

In the future, we expect that technological advances will continue to significantly impact the sensitivity, specificity, and scope of the NBS programs. Screening for other disorders such as hypothyroidism, blindness, and deafness has already started in Bahrain. A pilot study on metabolic disorders is also being planned.

## 6. Conclusion

This study shows that the incidence of SCD was <0.5% in 2010 among the study group, which is lower than that reported in previous studies conducted in Bahrain. This indicated a gradual decline in disease frequency, which may be due to the high awareness among the people of Bahrain that was a result of the educational and awareness campaign started approximately 20 years ago. With the continuation of education and awareness campaigns, carrier screening, and premarital services, we expect the number of affected newborns to reduce tremendously over the next few years.

## Figures and Tables

**Table 1:** Affected newborns with sickle cell disease in 2010.

Month	No. SCD	Total newborn	Percentage
January	4	968	0.41%
February	4	831	0.48%
March	4	854	0.46%
April	2	875	0.22%
May	6	944	0.63%
June	1	874	0.11%
Juley	5	996	0.5%
August	3	1007	0.29%
September	7	987	0.7%
October	8	1033	0.77%
November	3	958	0.3%
December	0	990	0

Total	47	11317	0.41%

**Table 2:** Affected newborns with sickle cell disease per year during 2007 to 2010.

	2007 and 2008		2009		2010	
	NO	%	NO	%	NO	%
SCD	123	0.7%	58	0.56%	47	0.41%
SCT	1969	11.3%	1605	15%	1673	14.7%
Normal	15283	88%	8585	83.7%	9644	85%

Total	17375		10248		11317	
